# Editorial: Inflammation in the CNS: Advancing the Field Using Intravital Imaging

**DOI:** 10.3389/fimmu.2017.01155

**Published:** 2017-10-02

**Authors:** Saparna Pai, Michael J. Hickey, Wolfgang Weninger

**Affiliations:** ^1^Australian Institute of Tropical Health and Medicine, James Cook University, Cairns, QLD, Australia; ^2^Immune Imaging Program, The Centenary Institute, Newtown, NSW, Australia; ^3^Sydney Medical School, University of Sydney, Sydney, NSW, Australia; ^4^Centre for Inflammatory Diseases, Monash University Department of Medicine, Monash Medical Centre, Melbourne, VIC, Australia; ^5^Discipline of Dermatology, University of Sydney, Sydney, NSW, Australia; ^6^Department of Dermatology, Royal Prince Alfred Hospital, Camperdown, NSW, Australia

**Keywords:** intravital imaging, leucocytes, inflammation, brain, central nervous system disorders

**Editorial on the Research Topic**

**Inflammation in the CNS: Advancing the Field Using Intravital Imaging**

Inflammation of the central nervous system (CNS) contributes to a diverse array of life-threatening and debilitating conditions. These include autoimmune conditions such as multiple sclerosis (MS), progressive degenerative conditions [Alzheimer’s disease (AD)], sterile inflammation as occurs in stroke/cerebral ischaemia, and inflammation stemming from parasitic, fungal, viral, and bacterial infections. Whilst recent developments have led to improved outcomes in some of these conditions, most notably MS (1), there remains concerns with these approaches (2). Furthermore, there is an increasing prevalence of AD and stroke among the ageing population in the developed world, whilst in sub-Saharan Africa, cerebral malaria remains a major cause of mortality. These factors mandate a greater understanding of the inflammatory mechanisms in the CNS associated with these conditions. As is the case with all inflammation, inflammatory responses in the CNS involve immune cell entry/migration, complex interplay between resident and circulating immune cells, parenchymal cells, the cellular constituents of the CNS microvasculature, and alterations in immune cell function.

Intravital or *in vivo* imaging has been a critical tool for understanding the mechanisms of inflammation throughout the body, including in the brain (3–5). Particularly, the advent of two-photon intravital microscopy (2P-IVM) has allowed researchers to directly examine the role of multiple immune cell populations in the initiation and regulation of inflammation within the CNS. 2P-IVM has become a critical tool not only for understanding the complex interplay between the cellular components of the immune system and how they act to provide protection against infection and injury but also how the dysregulation of these processes leads to disease.

Whilst application of intravital imaging to the CNS has been technically challenging, several issues have been systematically addressed over the years to facilitate generation of high-quality four dimensional (*x*,*y*,*z*,*t*) images. These advances have proved pivotal in understanding animal models of CNS inflammation such as EAE. Moreover, the combination of ongoing technical developments in imaging technologies, reporter mice, and novel fluorophores for detection of cellular signalling, in parallel with improved animal models of CNS disease, has meant that the understanding of inflammatory processes in this unique organ is better than ever before.

This *Frontiers Research Topic* brings together studies illustrating how imaging has advanced CNS inflammation and provides an overview of what parameters can be assessed using this approach. A key point that emerges from this collection is that intravital imaging has moved beyond simplistic descriptions of immune cell accumulation at inflamed sites—new approaches allow investigation of the molecular activities of these cells *in situ* in the CNS, in an ongoing inflammatory response, in unprecedented detail.

Activated T cells migrating in non-lymphoid organs play important homeostatic and pathological roles, including in the CNS. Gaylo et al. discuss the mechanisms that control T cell interstitial migration, emphasising that inflammation can cause changes in the composition of the extracellular milieu in which T cells navigate. They describe how one of the challenges of using imaging to assess T cell migration in the brain is the difference in the composition of the stromal and parenchymal CNS components relative to other peripheral tissues.

Lovelace et al. examine evidence linking dysregulation of the kynurenine pathway of tryptophan metabolism and the pathogenesis of MS, highlighting the role of mononuclear phagocytes in generation of neurotoxic metabolites *via* this pathway. Investigation of this pathway in CNS inflammation has involved a diverse range of imaging approaches as described in this article, including assessment of the blood–retinal barrier in the eye, MRI for non-invasive assessment of the blood–brain barrier, 2P-IVM for examination of T cell infiltration in EAE, and correlative scanning electron microscopy for assessment of cell–cell interactions in the brain.

Maysinger and Zhang define some of the emerging questions on the immunomodulatory effects of alimentary components, gut microbiota, and nanomaterials on microglial function and activation and discuss the use of bioluminescence-based platforms for these analyses.

Pietronigro et al. examine innovative 2P-IVM-based approaches for visualisation of the progression of amyloid beta deposition and alterations in microglial behaviour in a mouse model of AD. This approach has revealed previously unrecognised actions of neutrophils in amyloid plaques in the brain.

Radbruch et al. present two studies applying advanced *in vivo* fluorescence lifetime imaging to examine the brain in EAE (Radbruch et al.), and ageing and amyloid-related pathology (Radbruch et al.). These studies use markers to differentiate between myeloid cells and astrocytes. Importantly, the authors illustrate the capacity of contemporary imaging approaches to move beyond simply describing cell behaviour into understanding intracellular biochemistry and signalling of specific cell types in inflamed tissues, focussing on NAD(P)H oxidase (Nox) activity and Ca^2+^ signalling. In a separate article focussing on the remission phase of EAE, Radbruch et al. show that Nox activity remains elevated specifically in microglia. In contrast, under conditions of amyloid deposition, Nox activity is predominantly elevated in astrocytes (Radbruch et al.).

Fungal pathogens are an important cause of CNS pathology, particularly in immunocompromised individuals, although the mechanisms of fungal invasion of the brain are poorly understood. Shi and Mody describe the use of confocal intravital microscopy to investigate the dynamic interactions undergone by *Cryptococcus neoformans* in the CNS microvasculature, and the unusual nature of the neutrophil response to this infection.

Finally, Sonar and Lal provide an overview of the role of TNFSF receptor–ligand interactions in driving the pathogenesis of neuroinflammation and autoimmune disease in the CNS.

In summary, we anticipate that the collection of articles in this *Frontiers Research Topic* will provide researchers with a useful resource for understanding how imaging can be used to investigate the dynamics of CNS inflammation in its various forms.

## Author Contributions

SP: inviting contributions, handling and tracking submissions, setting timeline for research topic, acting as associate editor for selected manuscripts, inviting reviewers, coordinating with co-editors, and drafting and critically revising editorial. MH: inviting contributions, handling and tracking submissions, acting as associate editor for selected manuscripts, inviting reviewers, and drafting and critically revising editorial. WW: handling and tracking submissions, acting as associate editor for selected manuscripts, inviting reviewers, and drafting and critically revising editorial.

## Conflict of Interest Statement

The authors declare that the research was conducted in the absence of any commercial or financial relationships that could be construed as a potential conflict of interest.
